# Microscale tracking of coral-vibrio interactions

**DOI:** 10.1038/s43705-021-00016-0

**Published:** 2021-05-25

**Authors:** Assaf R. Gavish, Orr H. Shapiro, Esti Kramarsky-Winter, Assaf Vardi

**Affiliations:** 1 Department of Plant and Environmental Sciences, Weizmann Institute of Science, Rehovot, Israel; 2 Department of Food Quality and Safety, Agricultural Research Organization, Volcani Center, Rishon LeZion, Israel

**Keywords:** Microbiology, Microbial ecology

## Abstract

To improve our understanding of coral infection and disease, it is important to study host-pathogen interactions at relevant spatio-temporal scales. Here, we provide a dynamic microscopic view of the interaction between a coral pathogen, *Vibrio coralliilyticus* and its coral host *Pocillopora damicornis*. This was achieved using a microfluidics-based system facilitating control over flow, light and temperature conditions. Combined with time-resolved biochemical and microbial analyses of the system exudates, this approach provides novel insights into the early phases of a coral infection at unprecedented spatio-temporal resolution. We provide evidence that infection may occur through ingestion of the pathogen by the coral polyps, or following pathogen colonization of small tissue lesions on the coral surface. Pathogen ingestion invariably induced the release of pathogen-laden mucus from the gastrovascular cavity. Despite the high bacterial load used in our experiments, approximately one-third of coral fragments tested did not develop further symptoms. In the remaining two-thirds, mucus spewing was followed by the severing of calicoblastic connective tissues (coenosarc) and subsequently necrosis of most polyps. Despite extensive damage to symptomatic colonies, we frequently observed survival of individual polyps, often accompanied by polyp bail-out. Biochemical and microbial analyses of exudates over the course of symptomatic infections revealed that severing of the coenosarc was followed by an increase in matrix metaloprotease activity, and subsequent increase in both pathogen and total bacterial counts. Combined, these observations provide a detailed description of a coral infection, bringing us a step closer to elucidating the complex interactions underlying coral disease.

## Introduction

Coral reefs are currently undergoing an unprecedented decline driven by local and global changes to their environment.^1^ Reef building corals, commonly described as holobionts, form a complex relationship with photosynthesizing dinoflagellates (*Symbiodinium spp*.) and a consortium of microbial partners.^2^ Shifts in environmental conditions may lead to the breakdown of these symbiotic relations, often with catastrophic consequences for the coral colony. Such processes, collectively termed coral disease,^3,4^ may be manifested as a loss of the algal symbionts (coral bleaching),^2,5^ or as damage to the coral colony due to various forms of necrotic loss of coral tissue.^2^ On large scales, these processes may result in loss of coral cover, ultimately leading to the degradation of the reef structure and the loss of associated ecological and societal services.^4,6,7^

Many coral diseases are linked to specific pathogens whose abundance and virulence increase in response to environmental changes. Such changes may include nutrient loading, pollution, and temperature shifts.^8–11^ One of the best characterized coral diseases is the infection of the Indo-Pacific coral *Pocillopora damicornis* by the bacterial pathogen *Vibrio coralliilyticus.*^9,12,13^ The virulence of *V. coralliilyticus* is known to be positively correlated with increased temperatures.^9,14–17^ Increased ambient temperatures are further linked to accelerated vibrio growth rates,^9^ enhanced chemotaxis and chemokinesis,^17^ and secretion of matrix metalloproteases (MMPs).^18^ Nevertheless, a mechanistic understanding linking these traits to coral infection and disease progress is still lacking.

The majority of coral disease studies focus on monitoring coral colonies for the appearance of macroscopic signs of disease. These may include various forms of tissue discoloration, loss of the algal symbionts, or loss of tissue integrity.^19–21^ The focus on macroscale studies is derived to a large extent from the complexity of the coral holobiont,^21,22^ which makes it difficult to establish a tractable model system facilitating more detailed observations.^22,23^ Currently, the main available tool enabling to link a potential pathogen to the site of tissue damage and to the host response is histopathology.^23^ However, as such disease manifestations only appear at advanced stages of the infection process, their use as disease indicators fails to capture the early stages of pathogen colonization and disease initiation.^23^ We are thus lacking a mechanistic understanding of key steps in the infection process, including site of initial colonization, possible functions of specific disease markers such as MMP’s, or where bacterial chemotaxis may come into play. Furthermore, there are still major knowledge gaps in our understanding of coral response at the onset of pathogenic infection, including behavioral and physiological responses which may affect the ultimate outcomes of a bacterial infection.

Here, we present results from a microfluidic experimental platform for direct microscopic observation of coral-pathogen interactions over extended time periods (Supplementary Fig. 1). In contrast to a previously published system,^24^ the presented platform is designed to hold and observe small (5 mm) coral fragments, under controlled light, temperature, and flow conditions, while allowing the collection of system exudates for downstream microbial and biochemical analyses. Using this system allowed us to perform a series of controlled in vitro infection experiments, to specifically investigate the interaction between the bacterial pathogen *V. coralliilyticus* and its coral host *P. damicornis* at high spatio-temporal resolutions. Through these experiments we could follow the early stages of a coral infection, providing the most detailed description of a coral-pathogen interaction to date.

## Results

### Live imaging of coral infection

The robustness of the new microfluidic system was demonstrated by incubating healthy *P. damicornis* fragments under controlled environmental conditions (temperature, light, flow, and water quality) for periods of several days. Microscopic imaging allowed us to continuously monitor tissue integrity. Autofluorescence of coral green fluorescence protein (GFP), as well as of the chlorophyll of the algal symbionts, served as natural biomarkers for the wellbeing of the coral holobiont. No changes in coral morphology or behavior, and no extensive loss of algal symbionts (bleaching) were observed within the 48 h of incubation.

### Vibrio challenge of *P. damicornis* fragments

**Fig. 1:**
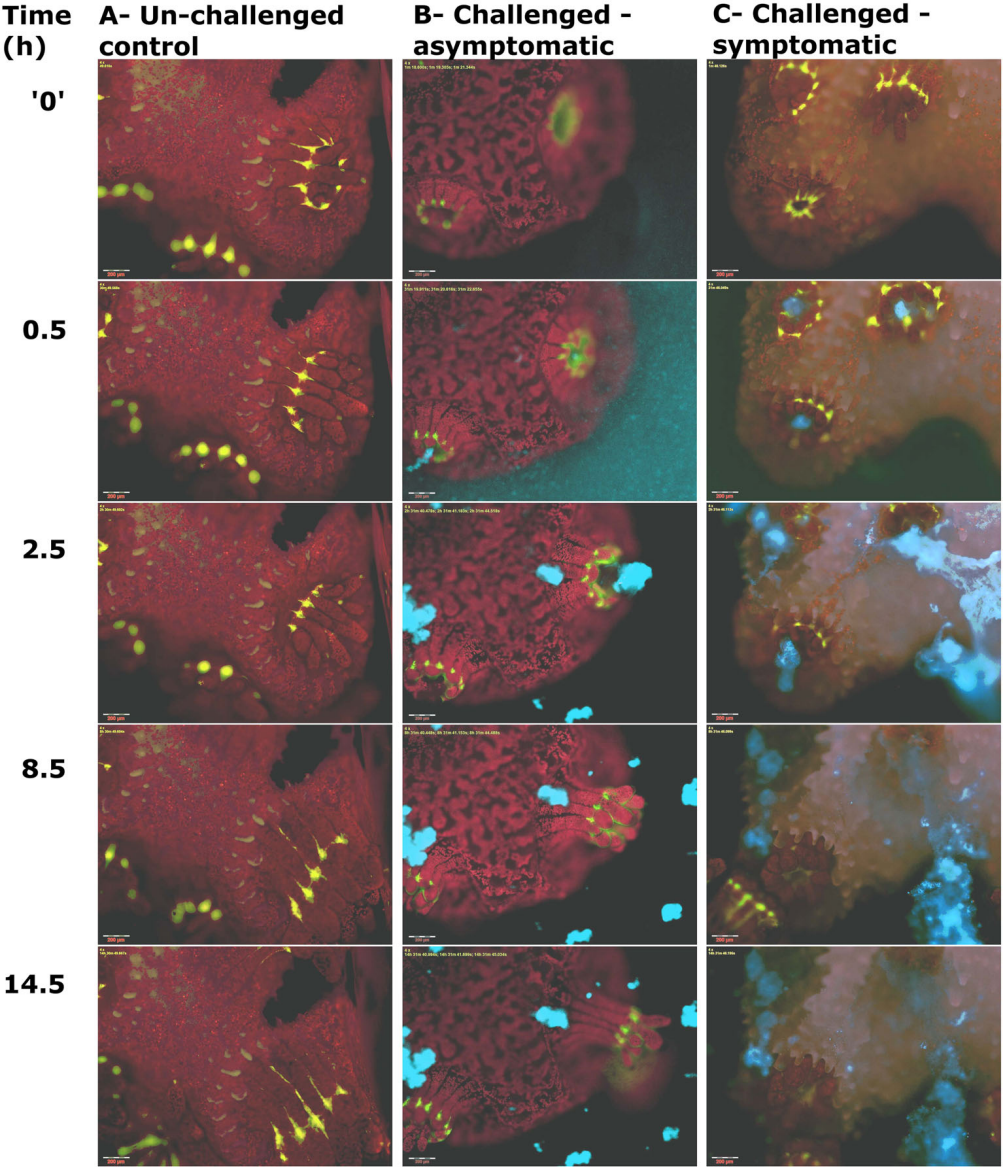
Timeline of a coral infection as revealed through live-imaging microscopy. **A** Microscopic view of an unchallenged control *P. damicornis* fragment showing coral GFP (green) and algal chlorophyll (red). All non-challenged fragments appeared healthy at the end of the experiment (here 14 h), with tentacles extended and no apparent bleaching or tissue loss. **B**, **C** Fragments challenged with *V. coralliilyticus* consistently displayed initial retraction of polyp tentacles (0–0.5 h). *V. coralliilyticus* accumulation (cyan) is observed primarily at the polyp pharynx, but not on other parts of the colony, within 0. 5 h of inoculation. This is followed by secretion of thick mucus, clearly visible due to the large numbers of Vibrio cells adhering to it (2.5–14 h). Challenged, asymptomatic fragments (**B**) rapidly recovered following mucus secretion, with tentacles extended and no visible loss of tissue, although mucus-flocs are still visible around the fragment at the end of the experiment. In symptomatic fragments (**C**), mucus secretion is followed by rapid loss of coenosarc tissue (2.5 h) and separation of polyps. Individual polyps then experienced one of three different fates. The majority of polyps were broken down, with loss of tissue integrity and GFP fluorescence (e.g. top right polyp in the presented sequence). The remaining polyps survived the infection, either attached to the coral skeleton (bottom left corner here) or detached from the calyx via polyp bail-out (here top left corner at time 2.5 h, migrating to the bottom of the frame in subsequent time points). Scale bars in all images are 200 μm.

**Fig. 2:**
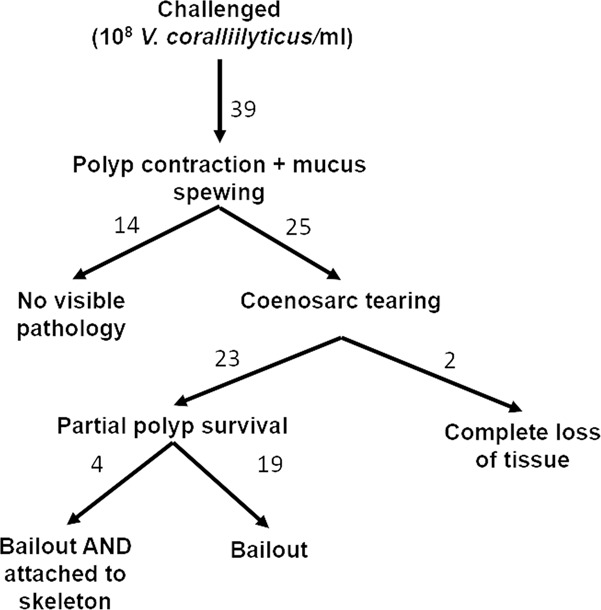
A roadmap of outcomes for *P. damicornis* fragments challenged by *V. coralliilyticus*. Of the 39 challenged fragments, 14 displayed no visible pathology following the infection challenge. The remaining 25 fragments were all symptomatic showing complete loss of colony integrity. In 2 of these fragments, a complete loss of all polyps was observed (experiment 6-1 in Table 1). In the remaining 23 fragments, some polyps survived the infection, either remaining attached to the skeleton or (more frequently) detached from the colony via polyp bailout. A summary of infection experiments and their outcomes is provided in Table 1.

No morphological or behavioral changes were observed in non-challenged control fragments in all experiments (Fig. 1A and Supplementary Video 1). In fragments inoculated with *V. coralliilyticus* transformed to express DsRed fluorescent protein (Table 1), accumulation of *V. coralliilyticus* cells was observed at the pharynx of all polyps in the microscope’s viewing field within 30 min from inoculation (Fig. 1B, C). This was followed by polyp contraction and discharge of Vibrio-laden mucus from the polyp pharynx. Following this stage, we consistently observed two possible outcomes to the vibrio challenge (Fig. 2). Of the 39 challenged fragments, 14 were asymptomatic. In these fragments, within 2–4 h from inoculation, DsRed labeled bacteria were no longer visible on the coral surface, although mucus flocs with attached bacteria were still present in the flow chamber (Fig. 1B). Tissue in asymptomatic fragments appeared morphologically intact, and within a few hours of inoculation polyps expanded, and no further pathology was observed (Supplementary Video 2).

**Table 1:** Summary of challenge experiments and outcomes.

Colony	Experiment	Fragments challenged	Observed Polyp fate		
			a	b	l
1	1-1	2	+	+	+
	1-2	2	+	−	−
	1-3	2	−	+	+
2	2-1	2	−	+	+
	2-2	2	−	+	+
	2-3	2	−	+	+
	2-4	2	+	+	+
3	3-1	2	+	−	−
	3-2	2	+	−	−
	3-3	2	+	−	−
4	4-1	2	−	+	+
	4-2	2	+	−	−
	4-3	2	+	−	−
	4-4	2	+	−	−
5	5-1	3	−	+	+
	5-2	3	−	+	+
	5-3	3	−	+	+
6	6-1	2	−	−	+

The fate of individual polyps at the end of the experiment is described as attached to the calyx (a), bailed-out (b), or lysed (l). For all experiments, the same number of fragments was used as unchallenged controls, with no pathology observed (Fig. 1A).

In 25 of the 39 fragments, defined as symptomatic, DsRed labeled *V. coralliilyticus* remained visible around the coral pharynx, and a clear pathology followed the initial response to the vibrio challenge. This was manifested as rapid lysis of the coenosarc tissue within 3 h of inoculation, effectively resulting in separation of the polyps and loss of the colonial form (Fig. 1C and Supplementary Video 3). The majority of the polyps in these experiments then underwent necrosis, manifested as visible loss of tissue integrity, accompanied by a gradual but constant decrease in the autofluorescence of the coral GFP (Green) most evident around the coral mouth (Fig. 1C, 0.5–8.5 h; Supplementary Video 3). No change was observed in the GFP of non-challenged controls or challenged, asymptomatic fragments (Fig. 1A, B and Supplementary Video 1 and 2).

Importantly, in 23 of the 25 symptomatic fragments, a number of polyps survived the infection despite extensive ceonosarc necrosis and loss of colony integrity. Surviving polyps either remained attached to the calyx, or, more frequently, were separated from the skeleton and underwent polyp bail-out,^24,26^ i.e. detachment of a live polyp from the calyx (Fig. 1C; Table 1; and Supplementary Video 3). The GFP signal in the surviving polyps appeared stable. Bailed-out polyps remained viable to the end of the experiment, as seen through their continual movement due to ciliary action. Significantly, bailed-out polyps retrieved from challenge experiments and transferred to a separate flow system remained viable for over 2 weeks from initial infection.

In a similar infection experiment using a DsRed labeled non-pathogenic *V. fischeri*, no retraction of polyps was observed following inoculation with 10^8^ cells/ml. *V. fischeri* accumulation at the pharynx was observed but to a lesser extent, possibly due to lower fluorescence signal of *V. fischeri* cells. Mucus secretion appeared greatly reduced compared to *V. coralliilyticus* inoculated fragments, with no evident necrosis (Supplementary Fig. 2).

### Lesions as hotspots of *V. coralliilyticus* colonization and infection

**Fig. 3:**
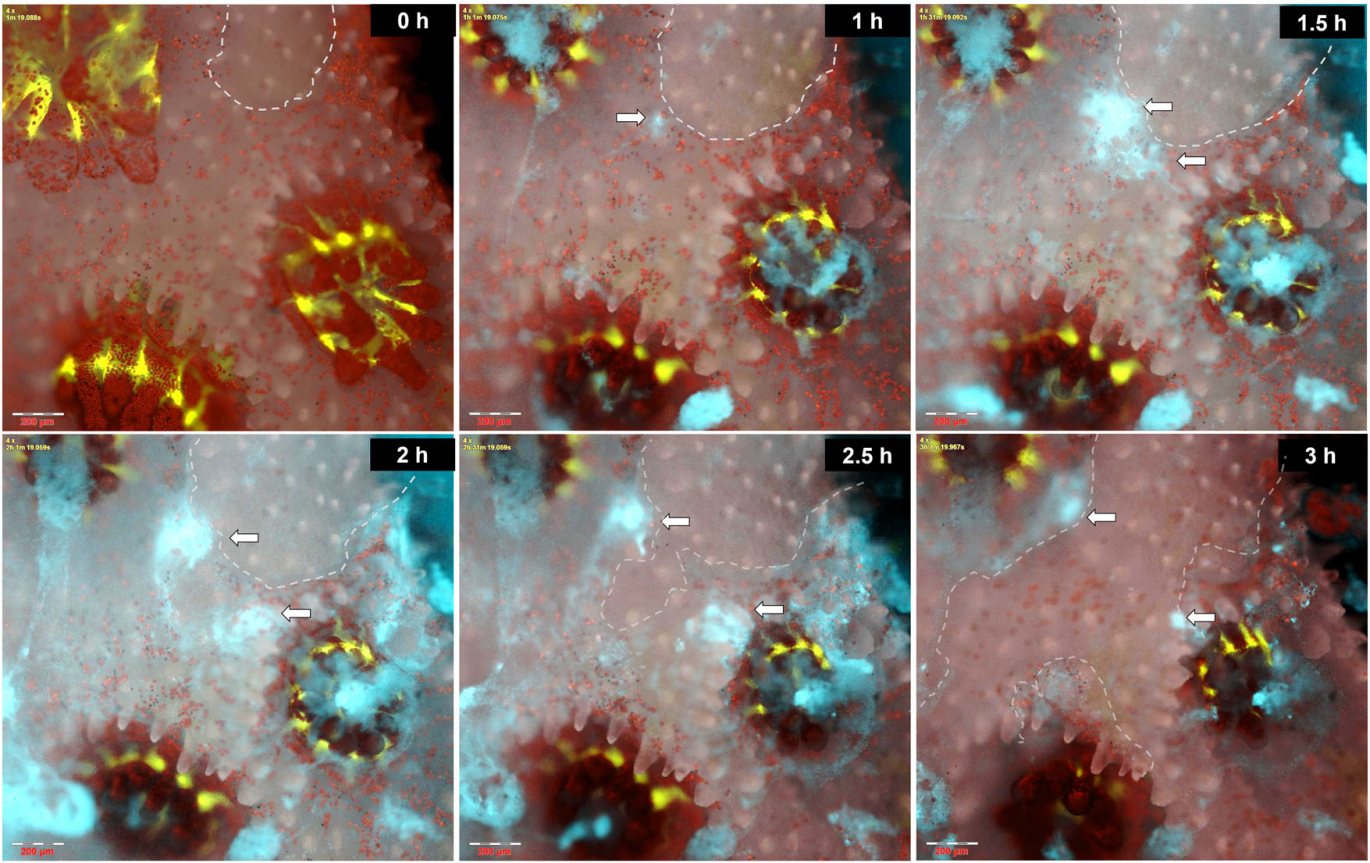
Tissue lesions allow rapid colonization and infection by *V. coralliilyticus*. A time series displaying *V. coralliilyticus* accumulation at the edge of a minor tissue lesion, with subsequent bacterial proliferation and extensive tissue necrosis. Time ‘0’ h–*P. damicornis* fragment immediately prior to inoculation. Coral GFP (green) and algal chlorophyll (red) are shown on the background of the colony’s skeleton (grey). Dashed line marks the borders of a small lesion approximately 300 μm in diameter. 1 h–*V. coralliilyticus* cells (cyan) settling at the lesion edge (arrow), are clearly distinct from pathogen-lades mucus flocs near polyp mouths. 1.5 h–3 h–further *V. coralliilyticus* accumulation at the torn tissue edge is followed by rapid lesion expansion and death of neighboring polyps. The complete sequence from this experiment is provided in Video S3. Scale bars are 200 μm.

We repeatedly observed that, in fragments with minor lesions in their coenosarc, rapid accumulation of *V. coralliilyticus* was visible at the lesion edge (Fig. 3 and Supplementary Video 4). Further bacterial accumulation, or possibly proliferation, was observed over the next hours, accompanied by tissue necrosis manifested as rapid tearing or degradation of the coenosarc tissue and death of neighboring polyps.

### Downstream analysis of system effluents facilitates quantitative description of the infection progress

**Fig. 4:**
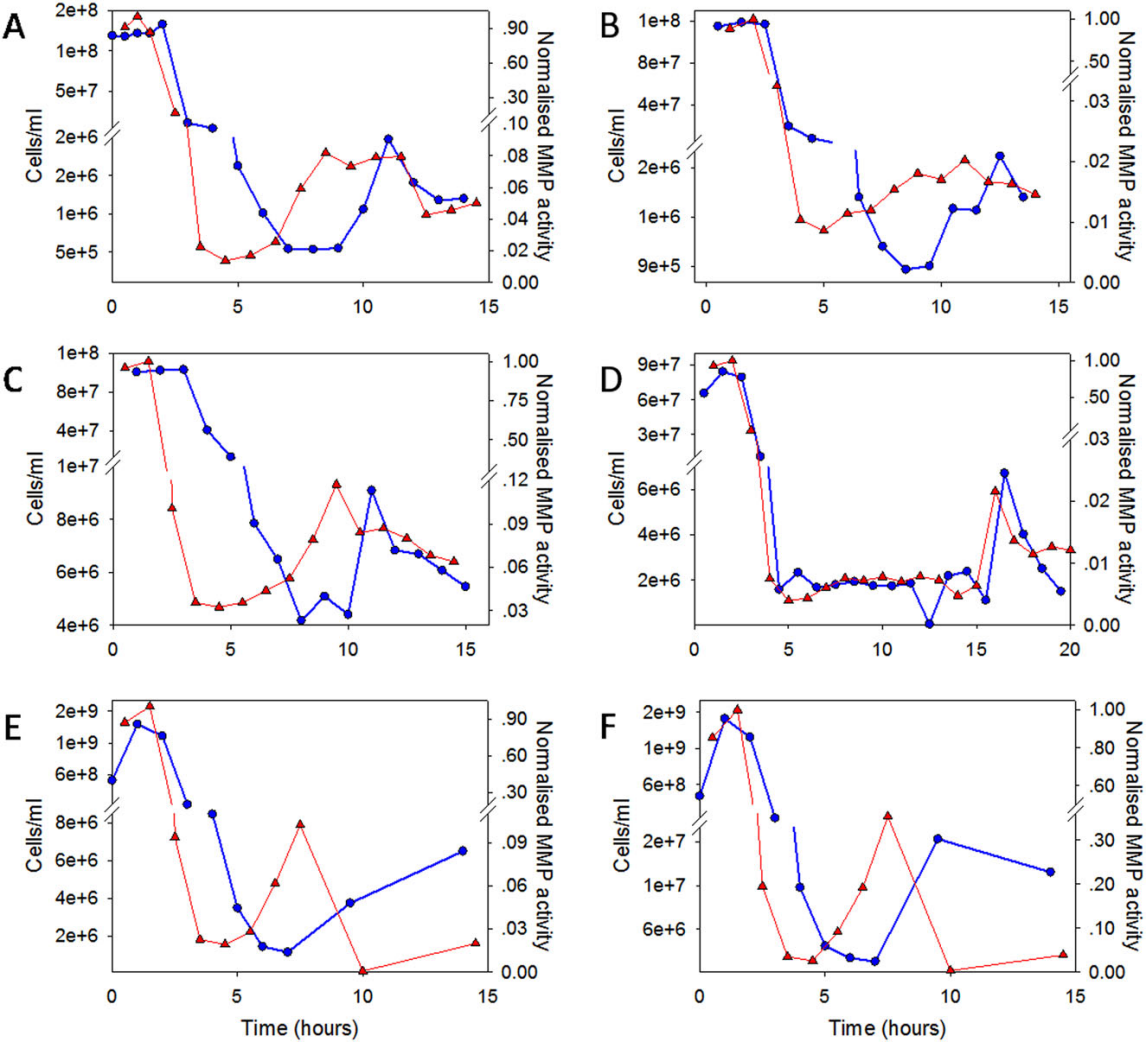
Changes in metalloprotease activity and *V. coralliilyticus* cell counts over the course of infection. MMP activity and total bacterial counts were quantified in time-resolved system exudates collected over the course of each infection experiment. Results are shown for five experiments. Panels (**A**–**D**) provide measurements obtained for challenged, symptomatic fragments obtained from three different colonies (A–colony 1; B, C–colony 2; D–colony 4). Panels (**E**, **F**) depict results from two fragments obtained from colony 6 and challenged in parallel in a single experiment. Red triangles–metalloprotease activity; Blue circles–Vibrio cell counts.

Additional insights into the infection process were gained by utilizing the system’s ability to collect exudates during the infection process, enabling a time-resolved measurement of *V. corlliilyticus* abundance and MMP activity in the system’s exudates (Fig. 4). For all *V. corlliilyticus*-challenged fragments, the highest values for both MMP activity and cell abundance were measured during the initial 2-h inoculation period. In the following hours, when incoming stream was changed to sterile seawater, bacterial abundance in the exudates decreased from 10^8^ cells/ml to ~10^6^ cells/ml, with a corresponding decrease in MMP activity (Fig. 4). In challenged, symptomatic fragments a subsequent rise of three to tenfold in MMP activity was observed 6 h or later post inoculation (Fig. 4). The observed increase in MMP activity was invariably followed by an increase in bacterial load of two to fivefold (Fig. 4). These late increases in bacterial abundance and MMP activity were not observed in control fragments or in the challenged, asymptomatic fragments (Supplementary Fig. 3).

Quantification of the fraction of DsRed positive cells out of the total bacteria in the exudates from one of the experiments (Supplementary Fig. 4) shows a decrease in their numbers following the inoculation period, reaching a minimum value of ~2 × 10^6^ cells/ml at 9.5 h post inoculation. DsRed labeled bacteria then rise to ~6 × 10^6^ at 10.5 h, and then decrease slightly, remaining relatively stable for the reminder of the experiment. Interestingly, the abundance of DsRed negative bacteria in the same experiment is seen to increase starting from 5.5 h post inoculation. Their value reached 4 × 10^6^ cells/ml at 10.5 h, comprising some 40% of total bacteria, but then decreased to 2 × 10^6^ by the end of the experiment.

### Fluorescence signals as markers of infection progression

Quantification of changes in fluorescence of both coral and bacteria over the infection period proved to be challenging due to the complex morphology of the coral fragment. Quantification of total DsRed fluorescence clearly depicted the increase in bacterial load during the inoculation period spanning the first 2 h of each experiment (Supplementary Fig. 5). In symptomatic fragments, a much smaller peak was often observed between 6 and 10 h following inoculation, roughly correlating with the observed increase in bacterial abundance in the exudates (Fig. 4), possibly marking the release of bacterial pathogens from the lysed tissue into the surrounding water. A gradual decay in coral GFP signal was evident 2–6 h following inoculation, possibly marking tissue degradation. No marked changes were observed in total chlorophyll fluorescence over the course of the experiments (Supplementary Fig. 5).

## Discussion

Coral disease progression is often studied at scales ranging from single colonies to the entire reef, thus overlooking the microscale processes governing host-pathogen interactions. While important insights into disease etiology have been gained by careful pathological and histological studies,^23,27–30^ such studies are mostly limited to snapshots of the disease process at acute and morphologically recognizable phases.^8,13,31–33^ Providing an in vivo trajectory of the sequence of events underlying the process of coral infection at relevant microscopic levels is important in the understanding of disease processes. The current work aims to bridge this gap, by studying infection of coral fragments under controlled conditions, at temporal and spatial scales relevant to the interactions occurring between a bacterial pathogen and its coral host.

We demonstrate for the first time that, during infection of coral fragments, *V. coralliilyticus* accumulates primarily at the polyp pharynx. This suggests an oral, and subsequently a gastrovascular, route of infection, in agreement with previous observations from experiments in detached, micropropagated polyps.^24^ Bacterial accumulation was not observed on the coenosarc surface except at tissue lesions. This is consistent with results described by Pollock et al.,^34^ using a GFP-labeled *V. coralliilyticus* P1. There, individual bacteria were shown to adhere to the surface of challenged *P. damicornis*, but no accumulation was evident on the coenosarc.^34^ Interestingly, that study did not report accumulation at the pharynx, possibly due to the higher magnification used, or because GFP-labeled cells were difficult to separate from the strong GFP background of the pharynx area.

In the current work, bacterial colonization of the colony surface was only observed around lesions in the coral tissue afflicted a priori to the challenge. This is consistent with similar associations between damaged tissues and coral disease reported previously.^23,27^ Accumulation at tissue lesions may be driven by bacterial chemotaxis toward signaling molecules (e.g. coral mucus) released by the damaged tissues,^17^ and facilitated by the loss of ciliary flows in the areas affected by tissue lesions.^36^ Our experiments thus further support previous results suggesting that coral vibriosis following infection by *V. coralliilyticus* proceeds through a gastrovascular route. We further provide a first direct demonstration that even minor lesions in the coenosarc may serve as a possible point of entry for *Vibrio* pathogens.

Beyond tracking of the bacterial pathogen, we observed patterns of coral behavior at the polyp scale, a subject that is rarely considered in the context of coral disease. We characterized the sequence of behavioral responses of the coral host following the bacterial challenge, and described specific processes occurring immediately following inoculation with pathogenic *V. coralliilyticus*. The coral polyps retracted into their calices and released copious amounts of mucus through their mouths. Polyp retraction is a universal response of corals to physical or environmental stress,^35–37^ indicating that the coral is sensing, and responding to, the presence of pathogens and/or their exudates. Indeed, as polyp retraction minimizes intake of water into its gastrovascular system, this behavior may provide a means for the coral to avoid further accumulation of pathogens in the gastric cavity. Similarly, mucus spewing is a common coral stress response to high loads of particulate matter in surrounding water.^38–40^ Thus, the sudden release of viscous, bacterial-laden mucus from the polyp mouth following bacterial challenge may be interpreted as an attempt by the coral to rid itself of the ingested pathogens, not unlike the coughing of phlegm during a lung infection in higher animals. These behaviors may thus be considered an important coral first line of defense against invading pathogens. This is further supported by the absence of these responses to similar concentrations of non-pathogenic *V. fischeri*.

In all *V. coralliilyticus-*challenged fragments, mucus spewing was invariably followed by a stretching of the inter-polyp coenosarc tissues. In symptomatic fragments, this ultimately led to tearing of the tissue and separation of adjacent polyps. Many of these isolated polyps survived, some even remaining attached to the skeleton while most underwent polyp bail-out.^24,26^ The survival of bailed-out polyps retrieved at the end of the experiment and maintained in filtered seawater further indicates that these polyps were able to overcome the invading pathogen. Polyp separation and bail-out thus provides the coral with an additional important defense layer, possibly enabling it to contain disease by “sacrificing” infected polyps, similar to plant hypersensitive response.^41^ The salvaging of individual polyps through polyp bail-out, recently shown to be a highly controlled process,^42,43^ provides an additional mechanism for preserving the coral genotype, as bailed-out polyps have been demonstrated to settle and regenerate into new colonies under favorable conditions.^24,26,42,44^

It is important to note that in all bacterial challenge experiments reported here, we used ~10^8^ *V. coralliilyticus* cells/ml. While this number is clearly unrealistic in ecological settings, at least in terms of planktonic bacteria, it is similar to cell numbers reported for many published laboratory experiments as required to induce quick and readily visible pathologies.^21,29,30,34^ While the need for such high density inoculum may signify a limitation of the short duration of our experiments, it is notable that previous experiments used similar *V. coralliilyticus* concentrations of between 10^7^ and 10^8^ cells/ml to induce infection in *P. damicornis.*^9,14,46^ Similarly, Ushijima et al.^47^ determined the infectious dose of *V. coralliilyticus* toward *Montipora* sp. to be between 10^7^ and 10^8^ cells/ml. These results suggest corals may be resistant to the low densities of planktonic *V. coralliilyticus* prevalent in the reef environment. An alternative route of infection in the field may occur via the ingestion of vibrio-laden marine snow or infected zooplankton^48,49^ delivering an infective dose of bacterial pathogens directly into the corals’ gastrovascular system.

A major question arising from our results relates to the function of MMP in the infection sequence. MMPs were previously suggested to be a key virulence factor of *V. coralliilyticus.*^18^ In our experiments, despite the exposure of all challenged corals to unnaturally high levels of MMPs secreted by the bacterial pathogens during inoculation step (Fig. 4A–F and Supplementary Fig. 4B), over 30% of these corals ultimately survived the infection (Table 1 and Fig. 2). Thus, under the conditions tested, MMP activity alone was not sufficient to kill all the corals. This is in agreement with a previous study where virulence of *V. coralliilyticus* was unaffected by the deletion of a gene encoding MMP production.^12^ In our experiments, an increase in MMP activity was observed at a relatively late stage of the infection, when polyps are likely to be already dying as indicated by the decay in GFP signal. The secretion of these metalloenzymes at this stage of the infection process may indicate their involvement in the breakdown of coral tissue, as a means for *V. coralliilyticus* to scavenge nutrients and essential metabolites from the decomposing tissues. Nevertheless, it cannot be ruled out that the increased MMP activity stems, at least in part, from the host tissues’ response to stress incurred following infection.^18,52^

An interesting observation arising from our experiments was that a large fraction (40%) of the microorganisms released in the exudates over the course of the infection were DsRed negative (Supplementary Fig. 2). This could have been explained by the loss of DsRed encoding plasmids from *V. coralliilyticus* transformants, but this is inconsistent with the rise in the DsRed positive fraction following the peak in bacterial abundance. An alternative explanation may be that additional bacterial populations, formerly part of the coral holobiont, are taking advantage of the lysis of the coral tissue and the associated abundance of nutrients. Future analysis of the bacterial community released from corals infected under similar settings may provide further insights into the identity and nature of such rogue members of the coral microbiome.

The lack of accumulation of *V. coralliilyticus* cells on the coral coenosarc appears to contrast with previous studies suggesting that chemotaxis toward coral surface mucus facilitates host-localization and colonization of the coral surface.^44–47^ Nevertheless, this view is challenged by a recent study demonstrating increased infectivity of *V. coraliilyticus* cells with impaired chemotaxis,^48^ similar to what was reported in *V. cholera*^49^ but differing from the fish pathgoen *V. anguillarum.*^50^ Bacterial chemotaxis occurs over relatively short distances (100’s of microns) and requires a stable and continuous gradient of the chemoattractant.^51^ As recently demonstrated, such conditions are not typically found near the surface of scleractinian corals.^36^ Ciliary flows exceeding 1 mm/s at the coral surface actively mix the coral’s boundary layer by creating vortices extending up to 2 mm into the surrounding water.^36^ These rapid currents, ten times the swimming speed of *V. coralliilyticus,*^17^ disrupt diffusion gradients that would otherwise develop in the coral’s boundary layer, while sweeping away any pathogens reaching the coral’s surface. Thus, ciliary flows are likely to prevent pathogens of scleractinian corals from chemotaxing toward their potential hosts. This is further supported by the accumulation of pathogens at tissue lesions, where ciliary flows are likely to be disrupted, as discussed above.

The question of chemotaxis is also relevant to the accumulation of pathogens at the coral pharynx. Significantly, while pathogen accumulation at lesion sites was only observed 45–60 min from inoculation, comparable accumulation at the coral pharynx is observed already 10–15 min following inoculation, suggesting different mechanism may be driving the two phenomena. We suggest that accumulation at the pharynx is driven by the active uptake of water into the coral’s gastrovascular system prior to polyp contraction, as part of ongoing feeding and gas exchange processes.^37,53–56^ Once inside the gastrovascular channels, where flow is likely to be laminar and cell boundaries within “easy reach”, chemotaxis may well play a part in targeted bacterial colonization of the gastrovascular mucus layer.

Our results revealed several hitherto unknown aspects of coral-vibrio interaction. We provide direct evidence *V. coralliilyticus* infection may be initiated through the gastrovascular system or at tissue lesions, raising interesting parallels with *Vibrio* infections in higher animals, including humans. We provide a first direct demonstration of behavioral defensive responses of the coral host, possibly serving to minimize pathogen intake at the onset of infection. The survival of individual polyps, including via polyp bail-out, may serve as a means of survival of the genotype following the demise of an infected colony. Future use of similar experimental designs will facilitate a more detailed understanding of the complex and ecologically important interactions occurring between corals and their bacterial pathogens at relevant scales.

## Methods

### Experimental setup

Microfluidic chambers were fabricated in-house as follows: A 5 × 1.5 cm slab was cut out of a 5 mm thick sheet of polydimethylsiloxane (PDMS) silicone elastomer (Sylgard® 184) using a utility knife. Four to six Ø8 mm wells were punched into the resulting slab using a biopsy punch of the same diameter, forming chambers of ~250 μl. Inlet and outlet holes were punched into opposing sides of each chamber using a 1 mm biopsy punch (Integra®, Fischer Scientific) (Fig. S1). The PDMS slab was then bonded to a glass microscope slide by exposing both glass and silicon surface to oxygen plasma for 1 min using a laboratory Corona Treater (Electro-Technic Products), attaching them and heating to 100 °C for 15 min. Each chamber was fitted with polyethylene inlet and outlet tubing (BPE-60, Instech Laboratories) (Fig. 1). The assembled device was placed on a temperature controlled microscope stage. Small *P. damicornis* fragments (3–5 mm) were placed in each chamber and chambers sealed with ApopTag® Plastic cover slips (Merck). Flow (2.6 ml h^−1^) was generated using a peristaltic pump (Ismatec) connected to the outlet tube. The input tube was connected to a flask containing FASW (0.22 μm). Inoculation was carried out by transferring the free end of the inlet tube to a flask containing FASW supplemented with 10^8^ of either *V. coralliilyticus* or *V. fischeri* for 2 h, and then transferring back to the FASW-containing flask.

The outlet stream from each chamber was continuously collected using a four channel fraction collector (Gilson Inc.) into 2 ml Eppendorf tubes, with tubes for each stream changed at 30 min intervals. Tubes were maintained in an aluminum tube rack placed in an ice bath to maintain contents at 0–1 °C. Every second tube was supplemented in advance with paraformaldehyde (PFA) to a final concentration of ~1%, enabling subsequent bacterial quantification using flow cytometry. Fractions from tubes without fixative were centrifuged, and supernatant used for quantification of MMP enzymatic activity.

### Coral collection and handling

All *P*. *damicornis* colonies used in this study were collected from a coral nursery located at a depth of 8 m off the pier of the Inter-University Institute, Eilat, Israel (Israel nature and parks authority permit No. # 2014/40327). Collected corals were maintained in an aquarium at the Weizmann Institute of Science. Small branch tips were clipped from the colonies and left in the main tank for recovery for at least 1 week. Prior to each experiment some fragments were transferred to a separate 4 l tank filled with FASW and incubated at 31 °C for a period of 3 days. The fragments were then transferred to the microfluidic device for microscopic observation. At the beginning of each experiment, prior to inoculation, fragments were acclimated on the stage for at least 3 h with a constant flow of filtered aquarium water.

### *Vibrio coralliilyticus* transformation to express DsRed

Infection experiments were performed using the *V. coralliilyticus* strain YB2 transformed by a plasmid encoding for a potent variant of DsRed2 fluorescent protein^25^ as described previously.^24^ For each experiment, DsRed labeled *V. coralliilyticus* were grown overnight from glycerol stock at 30 °C in Zobell Marine Broth. Bacteria were then centrifuged (3500 × *g*, 5 min) and re-suspended in FASW. Tubes were then incubated at 30 °C with no shaking to allow sinking of non-motile bacteria.

### Infection assays procedure

The general work flow and infection scheme are illustrated in Supplementary Fig. 1. Inoculation was carried out by flowing a suspension of DsRed labeled *V. coralliilyticus* or *V. fischeri* (~10^8^ cells/ml) into the chamber over a period of 2 h. Inlet flow was then switched to filtered aquarium water for the remaining incubation. Live imaging microscopy was carried out using a fully motorized inverted fluorescence microscope (Olympus IX81) equipped with a Coolsnap HQ2 CCD camera (Photometrics). Throughout the infection experiments, multichannel micrographs of the fragments were captured every 15 min at ×4 magnification. This enabled visualization of the coral-tissue GFP, zooxanthellae chlorophyll, and DsRed fluorescence, alongside a bright-field channel.

### Image analysis

Image analysis was carried out using imageJ (FIJI), by measuring mean gray intensity (in pixels) of the entire frame in each channel (GFP, Chlorophyll, and DsRed) captured at every time point, and plotting the change in intensity over the course of the experiment. This allowed us to follow changes in, e.g., coral GFP and algal chlorophyll fluorescence, as well as the abundance of DsRed labeled *Vibrio* cells, in the entire viewing field, providing a proxy for infection progression and coral health.

### Downstream exudate analysis

To couple the visual observations with direct microbial and biochemical measurements, each chamber’s effluents was continuously collected in a time resolved manner using a fraction collector and immediately cooled to between 0 and 1 °C (Fig. 1, step 5). Odd numbered fractions (1.3 ml) each were immediately fixed in 1% PFA in FASW. In total, 20 μl of each sample was diluted tenfold and stained with nucleic acid stain SYBR-gold (Invitrogen). Cell abundance was measured using a flow cytometer (iCyt Eclipse, excitation: 488 nm, emission: 500–550 nm). Even numbered fractions were collected with no fixation and filtered through 0.22 μm syringe filter (Millipore). Filtrate was used to estimate MMP activity using a specific fluorescent substrate (Calbiochem MMP-2/MMP-7 Substrate, Fluorogenic) in a microplate reader (Tecan Infinite® M200pro). Fluorescence (excitation: 325 nm, emission: 393 nm) was measure every 90 s at 30 °C for 40 min.

## Supplementary Information

Supplementary Video 1

Supplementary Video 2

Supplementary Video 3

Supplementary Video 4

Supplementary Information

## References

[1] Hoegh-Guldberg O, Bruno JF (2010). The impact of climate change on the world’s marine ecosystems. Science.

[2] Rosenberg E, Kellogg CA, Rohwer FL (2007). Coral microbiology. Oceanography.

[3] Rohwer, F. & Youle, M. *Coral Reefs in the Microbial Seas* (Plaid Press, 2010).

[4] Haas AF (2016). Global microbialization of coral reefs. Nat. Microbiol..

[5] Roth, E., Jeon, K. & Stacey, G. Homology in endosymbiotic systems: the term ‘symbiosome’. (1988).

[6] Zvuloni A (2009). Spatio-temporal transmission patterns of black-band disease in a coral community. PLoS ONE.

[7] Peters, E. C. in *Coral Reefs in the Anthropocene* 147–178 (Springer Press, 2015).

[8] Kushmaro A, Rosenberg E, Fine M, Ben Haim Y, Loya Y (1998). Effect of temperature on bleaching of the coral *Oculina patagonica* by Vibrio AK-1. Mar. Ecol. Prog. Ser..

[9] Ben-Haim Y (2003). *Vibrio coralliilyticus sp. nov*., a temperature-dependent pathogen of the coral *Pocillopora damicornis*. Int. J. Syst. Evol. Microbiol..

[10] Rosenberg E, Kushmaro A, Kramarsky-Winter E, Banin E, Yossi L (2009). The role of microorganisms in coral bleaching. ISME J..

[11] Kramarsky-Winter E, Downs C, Downs A, Loya Y (2009). Cellular responses in the coral *Stylophora pistillata* exposed to eutrophication from fish mariculture. Evol. Ecol. Res..

[12] Santos E (2011). Genomic and proteomic analyses of the coral pathogen *Vibrio coralliilyticus* reveal a diverse virulence repertoire. ISME J..

[13] Ben Haim, Y. & Rosenberg, E. A novel Vibrio sp. pathogen of the coral *Pocillopora damicornis*. *Mar. Biol*. **141**, 47–55 (2002).

[14] Vidal-Dupiol, J. et al. Coral bleaching under thermal stress: putative involvement of host/symbiont recognition mechanisms. *BMC Physiol.* **9**, 14 (2009). 10.1186/1472-6793-9-14 PMC2728513 19653882

[15] Zvuloni A, Artzy-Randrup Y, Katriel G, Loya Y, Stone L (2015). Modeling the impact of white-plague coral disease in climate change scenarios. PLoS Comput. Biol..

[16] Wright RM (2017). Intraspecific differences in molecular stress responses and coral pathobiome contribute to mortality under bacterial challenge in Acropora millepora. Sci. Rep..

[17] Garren M, Son K, Tout J, Seymour JR, Stocker R (2016). Temperature-induced behavioral switches in a bacterial coral pathogen. ISME J..

[18] Sussman M (2009). Vibrio zinc-metalloprotease causes photoinactivation of coral endosymbionts and coral tissue lesions. PLoS ONE.

[19] Rosenberg, E. & Kushmaro, A. in *Coral Reefs: An Ecosystem in Transition* 451–464 (Springer, 2011).

[20] Bourne D (2009). Microbial disease and the coral holobiont. Trends Microbiol..

[21] Pollock FJ, Morris PJ, Willis BL, Bourne DG (2011). The urgent need for robust coral disease diagnostics. PLoS Pathog..

[22] Weis V, Davy S, Hoegh-Guldberg O, Rodriguez-Lanetty M, Pringle J (2008). Cell biology in model systems as the key to understanding corals. Trends Ecol. Evol..

[23] Work, T. & Meteyer, C. To understand coral disease, look at coral cells. *EcoHealth* **11, **610–618 (2014). 10.1007/s10393-014-0931-1 24723160

[24] Shapiro OH, Kramarsky-Winter E, Gavish AR, Stocker R, Vardi A (2016). A coral-on-a-chip microfluidic platform enabling live-imaging microscopy of reef-building corals. Nat. Commun..

[25] Dunn AK, Millikan DS, Adin DM, Bose JL, Stabb EV (2006). New rfp- and pES213-derived tools for analyzing symbiotic *Vibrio fischeri* reveal patterns of infection and lux expression in situ. Appl. Environ. Microbiol..

[26] Sammarco PW (1982). Polyp bail-out: an escape response to environmental stress and a new means of reproduction in corals. Mar. Ecol. Prog. Ser. Oldendorf.

[27] Work TM, Aeby GS (2011). Pathology of tissue loss (white syndrome) in *Acropora* sp. corals from the Central Pacific. J. Invertebr. Pathol..

[28] Work TM, Aeby GS (2006). Systematically describing gross lesions in corals. Dis. Aquat. Org..

[29] Ainsworth T, Fine M, Roff G, Hoegh-Guldberg O (2008). Bacteria are not the primary cause of bleaching in the Mediterranean coral *Oculina patagonica*. ISME J..

[30] Ainsworth TD, Fine M, Blackall LL, Hoegh-Guldberg O (2006). Fluorescence in situ hybridization and spectral imaging of coral-associated bacterial communities. Appl. Environ. Microbiol..

[31] Boyett HV, Bourne DG, Willis BL (2007). Elevated temperature and light enhance progression and spread of black band disease on staghorn corals of the Great Barrier Reef. Mar. Biol..

[32] Gignoux-Wolfsohn S, Marks CJ, Vollmer SV (2012). White band disease transmission in the threatened coral, *Acropora cervicornis*. Sci. Rep..

[33] Kaczmarsky LT (2006). Coral disease dynamics in the central Philippines. Dis. Aquat. Org..

[34] Horridge GA (1957). The co-ordination of the protective retraction of coral polyps. Philos. Trans. R. Soc. Lond. B Biol. Sci..

[35] Katz SM, Pollock FJ, Bourne DG, Willis BL (2014). Crown-of-thorns starfish predation and physical injuries promote brown band disease on corals. Coral Reefs.

[36] Shapiro OH (2014). Vortical ciliary flows actively enhance mass transport in reef corals. Proc. Natl Acad. Sci. USA.

[37] Gladfelter E (1983). Circulation of fluids in the gastrovascular system of the reef coral *Acropora cervicornis*. Biol. Bull..

[38] Patterson MR (1992). A chemical engineering view of cnidarian symbioses. Am. Zool..

[39] Lewis J, Price W (1975). Feeding mechanisms and feeding strategies of Atlantic reef corals. J. Zool..

[40] Brown BE, Bythell JC (2005). Perspectives on mucus secretion in reef corals. Mar. Ecol. Prog. Ser..

[41] Zetsche E, Baussant T, Meysman FJ, van Oevelen D (2016). Direct visualization of mucus production by the cold-water coral *Lophelia pertusa* with digital holographic microscopy. PLoS ONE.

[42] Lam E, Kato N, Lawton M (2001). Programmed cell death, mitochondria and the plant hypersensitive response. Nature.

[43] Chuang PS, Mitarai S (2020). Signaling pathways in the coral polyp bailout response. Coral Reefs.

[44] Kvitt H (2015). Breakdown of coral colonial form under reduced pH conditions is initiated in polyps and mediated through apoptosis. Proc. Natl Acad. Sci. USA.

[45] Garren M (2014). A bacterial pathogen uses dimethylsulfoniopropionate as a cue to target heat-stressed corals. ISME J..

[46] Garren M, Azam F (2012). Corals shed bacteria as a potential mechanism of resilience to organic matter enrichment. ISME J..

[47] Banin E, Israely T, Fine M, Loya Y, Rosenberg E (2001). Role of endosymbiotic zooxanthellae and coral mucus in the adhesion of the coral-bleaching pathogen *Vibrio shiloi* to its host. FEMS Microbiol. Lett..

[48] Meron D (2009). Role of flagella in virulence of the coral pathogen *Vibrio coralliilyticus*. Appl. Environ. Microbiol..

[49] Certner RH, Dwyer AM, Patterson MR, Vollmer SV (2017). Zooplankton as a potential vector for white band disease transmission in the endangered coral, *Acropora cervicornis*. PeerJ.

[50] Ushijima B (2014). *Vibrio coralliilyticus* strain OCN008 is an etiological agent of acute *Montipora* white syndrome. Appl. Environ. Microbiol..

[51] Butler SM, Camilli A (2005). Going against the grain: chemotaxis and infection in *Vibrio cholerae*. Nat. Rev. Microbiol..

[52] Stocker R (2015). The 100 µm length scale in the microbial ocean. Aquat. Microb. Ecol..

[53] Csaszar NBM, Seneca FO, van Oppen MJH (2009). Variation in antioxidant gene expression in the scleractinian coral Acropora millepora under laboratory thermal stress. Mar. Ecol. Prog. Ser..

[54] Agostini S (2012). Biological and chemical characteristics of the coral gastric cavity. Coral Reefs.

[55] Houlbrèque, F., Rodolfo‐Metalpa, R. & Ferrier‐Pagès, C. Heterotrophic nutrition of tropical, temperate and deep‐sea corals. *Dis. Coral* 150–163 (2015).

[56] Sorokin YI (1973). Trophical role of bacteria in the ecosystem of the coral reef. Nature.

