# Supplementary material for: Microscale tracking of coral-vibrio interactions
Source: ISME Commun. 2021 May 25;1:18. doi: 10.1038/s43705-021-00016-0 (PMC9723675; doi:10.1038/s43705-021-00016-0)
Supplement: Supplementary file 5 — Supplementary Information [file 43705_2021_16_MOESM5_ESM.pdf]

Supplementary information for

Microscale tracking of coral-vibrio interactions

Assaf R. Gavish[1†], Orr H. Shapiro[1,2†,*], , Esti Kramarsky-Winter[1] and Assaf Vardi[1*]

[1]Department of Plant and Environmental Sciences, Weizmann Institute of Science, Rehovot, Israel. [2]Department of Food Quality and Safety, Agricultural Research Organization, Volcani Center, Rishon LeZion, Israel.

[†]These authors contributed equally to the work

*Corresponding authors: orr@agri.gov.il, assaf.vardi@weizmann.ac.il

The microfluidic experimental platform used here has several features distinguishing it from the previously published coral-on-a-chip system[24]. The larger chamber volume and higher flow rates of the current system facilitate the incubation of small coral fragments, preserving the colonial morphology of the coral colony. This system allows the incubation and tracking of up to 4 individual coral fragments in separate chambers, facilitating flexible experimental design. Moreover, it allows the continuous collection of exudates of the system for downstream analysis. The experimental workflow is described below.

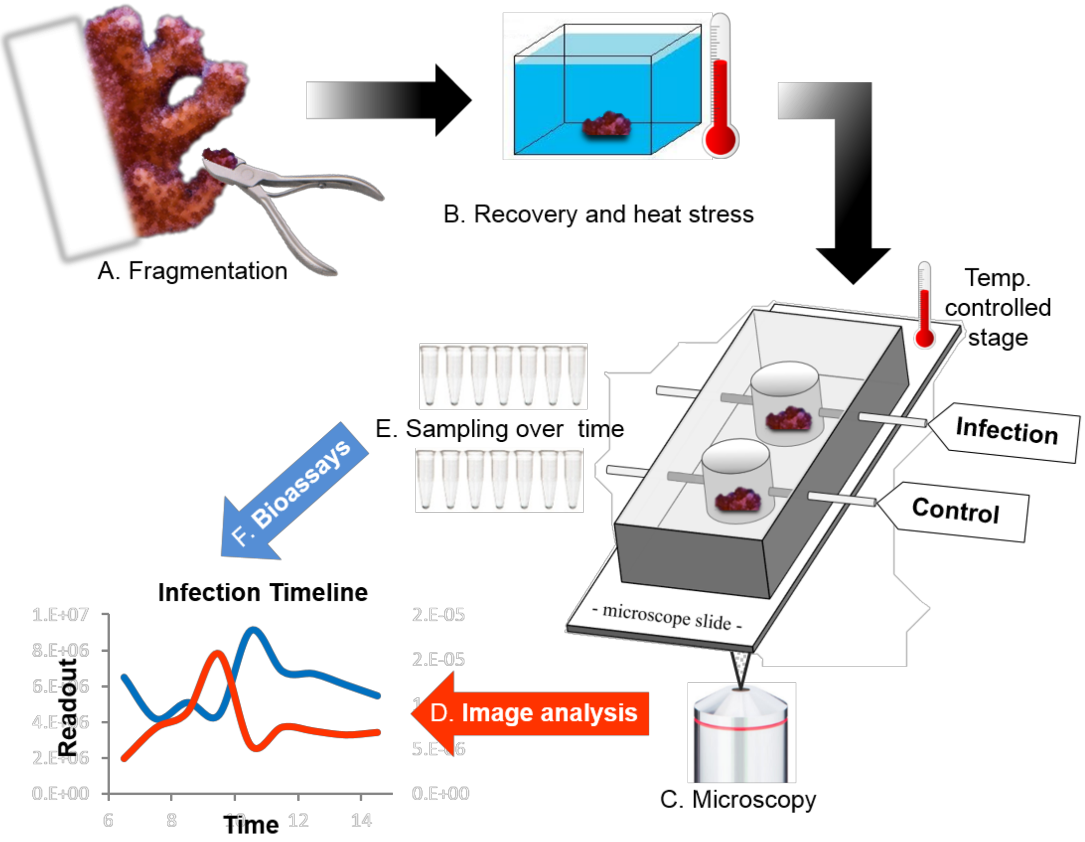

Supplementary Figure 1: Experimental work-flow for microfluidic coral infection experiments.

A, B. Small coral fragments (~3-5 mm), clipped from the branch tips of a *P. damicornis* colony, are kept in the main tank for recovery. Fragments are incubated for 3 days in a small (3.5 L) temperature-controlled tank filled with filtered aquarium water. Heat stress is induced by setting tank temperature to 30oC. C. Fragments are transferred to the microfluidic device placed on a temperature-controlled microscope stage. Infection is initiated by introducing DsRed-labelled *V. coralliilyticus* cells at desired duration and concentration through the inflow. Infection progress is tracked using epifluorescence and light microscopy at set intervals. D. Image analysis is used to quantify signal intensity and localization in all channels throughout the infection period. E. An automated fraction collector is used to sample flow through at set intervals throughout the experiment. Collected fractions are immediately cooled to below 2°C, with or without addition of fixative, for subsequent analysis. F. Collected fractions are analyzed by various bioassays, enabling correlation of microscopic observations and downstream analysis.

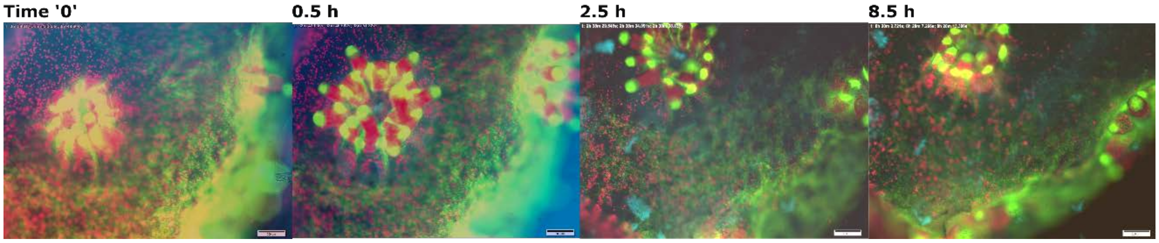

Supplementary Figure 2: Challenge experiment with non-pathogenic *Vibrio fischeri*
Starting at time '0', 10^8 cells/ml of DsRed-labelled *V. fischeri* cells were inoculated into the flow chamber for 2 h and inflow was then changed to sterile artificial seawater similar to experiments with *V. coralliilyticus.* Some accumulation of DsRed labelled cells, possibly attached to mucus flocs, is seen at and around the coral pharynx at 2.5 h post inoculation, and mucus flocs can still be seen at 8.5 h post inoculation. Despite the high bacterial load, no polyp retraction was observed at any time during the experiment, and no pathology was observed over the incubation period. *V. fischeri* challenge was performed on 2 fragments obtained from colony 6, in parallel with two fragments challenged with *V. coralliilyticus* that underwent complete necrosis (experiment 6-1 in Table 1 in the main text).

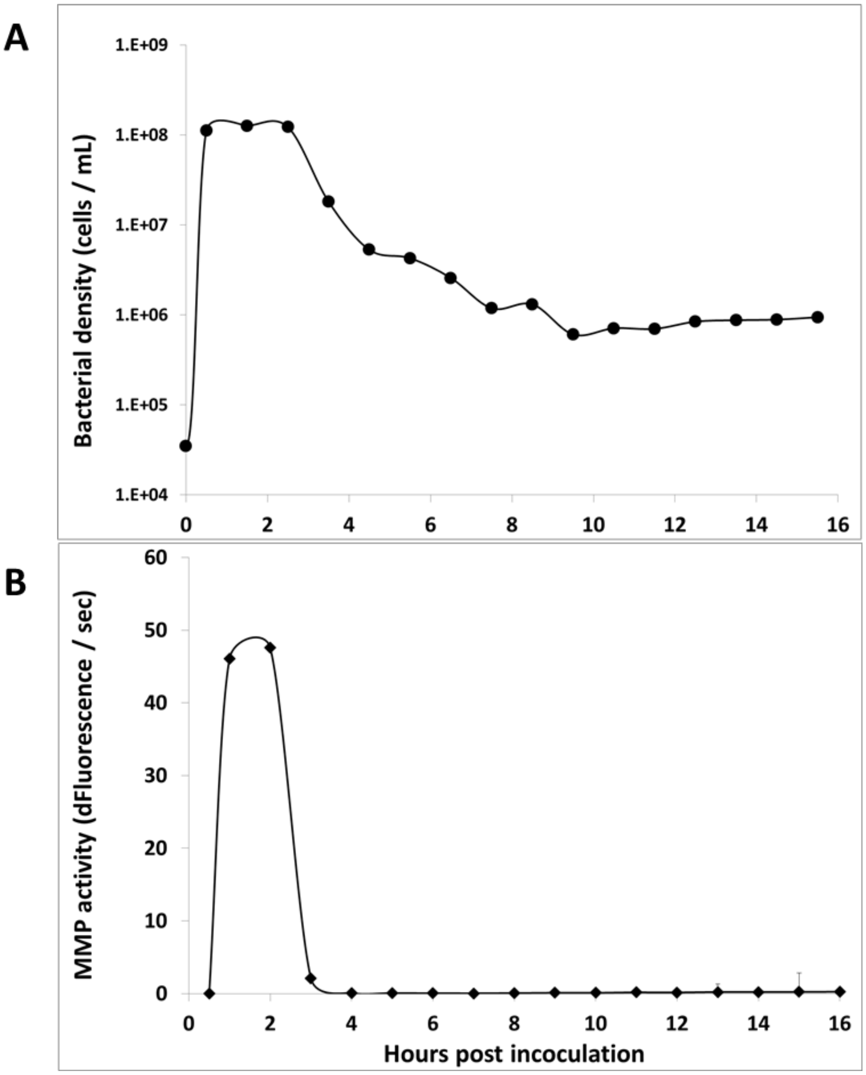

Supplementary Figure 3: Downstream analysis for a challenged, non-symptomatic *P. damicornis* fragment.

A. Changes in total bacterial counts in the chamber exudates over the course of the experiment. Bacterial density in the exudates gradually decreases from 10^8 cells/ml to 10^6 cells/ml over the 8 h following the inoculation period, and remains at that level for the duration of the experiment. **B.** Changes in Metalloprotease activity in the chamber exudates over the course of the experiment. MMP activity peaks during the 2 h inoculation period, and then drops to near ‘0’ for the duration of the experiment. Note that MMP units are arbitrary, and cannot be used to compare activity levels between different experiments.

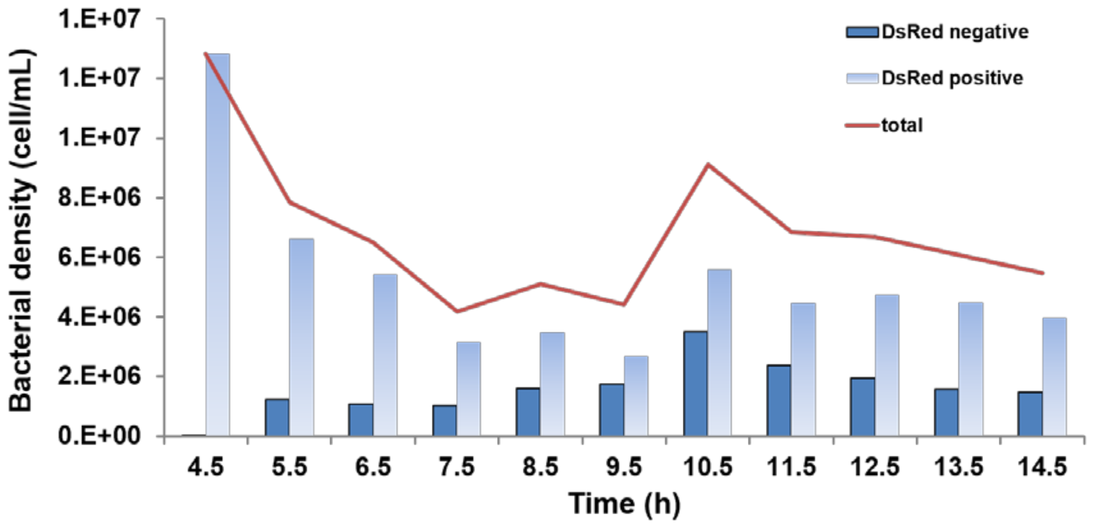

Supplementary Figure 4: Total and DsRed-labelled bacteria in the system's effluents over the course of a single infection process (the same process as presented in Fig. 4B).

The fraction of DsRed-labeled cells gradually decreases from over 99% at the end of the inoculation period to just over 60% during peak bacterial abundance at 10.5 h post inoculation, and then gradually rises to ~75% over the following hours. Values are mean of three technical replicates.

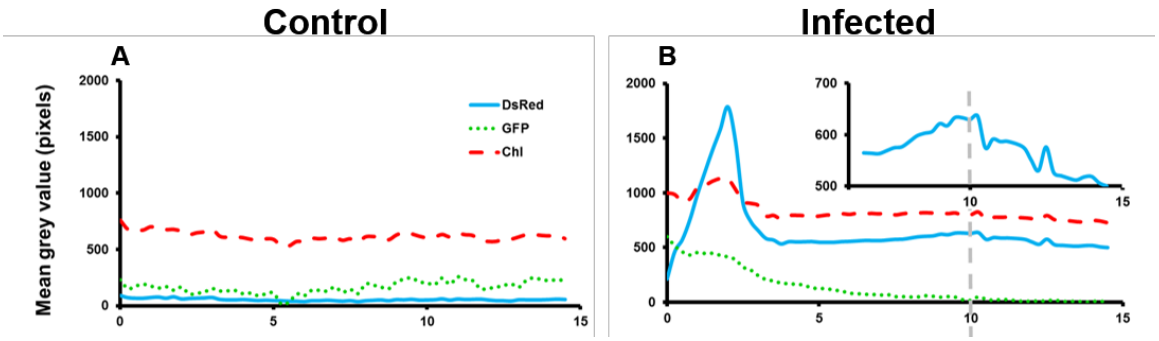

Supplementary Figure 5: Changes in fluorescence signals of the different components of the holobiont over the course of a challenge experiment. In the control experiments no changes in fluorescence in DsRed (bacteria), GFP (coral tissues) or chlorophyll (algal symbionts) were observed. In the challenge experiments, a rapid increase and subsequent decrease in DsRed fluorescence is observed over the first 4 h of the experiment, corresponding to the loading and washing out of labelled *V. corallilyticus.* A slight increase at around 10 h following inoculation (inset) may mark the release of newly formed pathogens from the lysed coral tissue. Chlorophyll fluorescence is observed to slightly peak over the same period, possibly due to leaking signal from the DsRed label, but then remains stable for the duration of the experiment. GFP fluorescence gradually decreases following over the course of the experiment, with near complete loss of signal at approximately 10 h from inoculation.

Captions for supplementary videos:

Video 1: **Control (unchallenged) *P. damicornis* fragment** observed for a period of 15 h, with polyps extended and motile and no visible loss of chlorophyll or GFP fluorescence.

Video 2: **Challenged, asymptomatic *P. damicornis* fragment**. DsRed-labelled *V. coralliilyticus* cells are seen to accumulate at the polyps' pharynx, as well as around the polyp base, during inoculation, but the coral appears to rid itself of the majority of pathogens and appears to survive intact over the course of the experiment.

Video 3: **Challenged, symptomatic *P. damicornis* fragment**. *V. coralliilyticus* accumulate at the polyps' pharynx, followed by copious spewing of pathogen-laden mucus from the coral gut. Coenosarc tearing results in separation of the polyps and breakdown of the colonial form. Separated polyps follow one of three fates, death, survival or polyp bailout.

Video 4: **Challenged, symptomatic *P. damicornis* fragment with minor lesion**. Here *V. coralliilyticus* cells are seen to accumulate at the edge of a 300 μm lesion, likely inflicted during handling of the coral fragment. Lesion colonization results in rapid proliferation of the bacteria and degradation of the coenosarc, followed by rapid demise of adjacent polyps.
